# Advancing Diagnosis of Current Hepatitis C Virus Infection: A Key to Hepatitis C Elimination in the United States

**DOI:** 10.1093/infdis/jiae127

**Published:** 2024-03-11

**Authors:** Saleem Kamili, Carolyn Wester

**Affiliations:** Division of Viral Hepatitis, Centers for Disease Control and Prevention, Atlanta, Georgia, USA; Division of Viral Hepatitis, Centers for Disease Control and Prevention, Atlanta, Georgia, USA

**Keywords:** hepatitis C, hepatitis C virus, diagnosis, elimination, viral hepatitis

## Abstract

More than 2 million adults have hepatitis C virus (HCV) infection in the United States, and new infections continue to increase. Without treatment, HCV infection can lead to advanced liver disease and death. Treatment is recommended for nearly everyone with hepatitis C, resulting in a cure in >95% of people treated and raising the possibility of hepatitis C elimination. Testing is the first step to accessing life-saving treatment. The Centers for Disease Control and Prevention recommends hepatitis C screening for all adults, all pregnant persons, and anyone with risk; yet about one-third of people with hepatitis C remain unaware of their infection. Testing begins with a hepatitis C antibody test, followed, when reactive, by a nucleic acid test to detect HCV RNA. This antibody-first, 2-step testing strategy misses early infections and can result in incomplete diagnoses. Advancements in hepatitis C diagnostics and the US regulatory landscape have created an opportunity to include viral-first testing strategies and improve hepatitis C diagnosis. This journal supplement features 8 articles detailing challenges and opportunities for improving hepatitis C diagnostics in support of advancing hepatitis C elimination in the United States.

More than 2 million adults in the United States are estimated to have hepatitis C virus (HCV) infection [1]. New infections continue to increase primarily in association with injection drug use; nearly 70 000 estimated cases of acute hepatitis C occurred in 2021 alone [2]. More than half of new infections progress to chronic infection [3]. Without treatment, HCV infection can lead to advanced liver disease, liver cancer, and death [4]. Safe and effective treatment has been available since 2014 that cures more than 95% of all treated persons, preventing downstream health complications, halting transmission, and raising the possibility of hepatitis C elimination [5]. Consistent with World Health Organization global hepatitis C elimination goals, the United States has called for a 65% reduction in hepatitis C–related deaths by 2030 [6].

Testing is the first step to accessing life-saving treatment; however, about one-third of people with hepatitis C in the United States are unaware of their infection [1]. The Centers for Disease Control and Prevention (CDC) recommends hepatitis C screening for all adults at least once, all pregnant persons during every pregnancy, and all persons with risk, including periodic testing if risk persists [7]. Current testing guidance for clinicians and laboratorians begins with a hepatitis C antibody (anti-HCV) test followed, when reactive, by a nucleic acid test (NAT) to detect HCV RNA to distinguish between past and current infection [8]. The current testing tools and diagnostic sequence has several limitations: first, it takes an average of 7–8 weeks after HCV infection for a person to have a reactive HCV antibody test [9], resulting in missed diagnoses of current HCV infection; second, the 2-step testing approach can lead to incomplete diagnoses when HCV RNA testing is not automatically conducted for all anti-HCV reactive samples; and third, the lack of point-of-care (POC) viral tests in the United States misses opportunities for rapid diagnosis and treatment initiation, which is particularly important in populations that are not engaged in longitudinal care and often heavily affected by hepatitis C. Achieving HCV elimination in the United States requires increased access to simple, accurate, affordable hepatitis C diagnostic tests and treatments in a variety of HCV screening, diagnosis, and treatment settings.

Fortunately, advancements in the diagnostic and regulatory landscape have created an opportunity to improve hepatitis C diagnosis. In November 2021, the US Food and Drug Administration (FDA) reclassified hepatitis C diagnostic tests from class III devices to class II devices with special controls (510k). This reclassification provides a new, lower-barrier opportunity for manufacturers to introduce new hepatitis C diagnostic tools for FDA review, including a NAT for HCV RNA detection in a POC format and hepatitis C core antigen (HCV cAg) assay, both of which are currently in use in a number of countries outside the United States.

With CDC support, the Association of Public Health Laboratories (APHL) held a 2-day convening of key stakeholders and subject matter experts in late 2021 to identify high-priority diagnostic tools needed to advance diagnosis of HCV infection and linkage to treatment in a range of clinical and nonclinical settings [10]. This supplement features 8 articles by subject matter experts participating in the APHL convening, detailing challenges and opportunities for improving hepatitis C diagnostics in support of advancing hepatitis C elimination in the United States.

The article by Feld [11] provides an overview addressing what is needed to move toward single-step diagnosis of current HCV infection and highlights challenges with the current 2-step HCV diagnosis. The use of HCV cAg and a POC HCV RNA test are also discussed in the article. Matching the right testing approach to appropriate settings will be needed until the ultimate goal of having a single test for HCV diagnosis is achieved. The author further summarizes the role of new technologies and discusses simple strategies using existing tools for simplification of diagnosis, which have the potential to improve linkage to care and treatment of HCV-infected individuals.

The article by Terrault [12] discusses the implications of the down-classification of HCV diagnostics from class III to class II for screening and diagnosis of current HCV infection. The ease of regulatory hurdles posed by higher classification and the importance of down-classification of HCV diagnostics in the potential development of new diagnostic tools and subsequent formulation of simplified diagnostic algorithms is also discussed. The author further discusses the remaining issues with the down-classification, especially its effect on the approval of HCV cAg tests in the United States.

Page and Feinberg [13] discuss what is working and what is needed in their article on what diagnostic tools are required to advance diagnosis of current HCV infection in outreach and nonclinical settings. They describe the characteristics of optimal diagnostic tools and outline the requirements of a Clinical Laboratory Improvement Amendments (CLIA) waiver for a diagnostic test issued by FDA. The need for alternate sample collection methods that do not require phlebotomy, such as dried blood spots, and a POC HCV RNA test for expediting and facilitating a “test and treat” approach are further discussed in this article.

The article by Handanagic et al [14] describes lessons learned from several countries that have used decentralized and integrated models to increase access to HCV testing toward their efforts to eliminate hepatitis C. The authors discuss, as an example, how the country of Georgia was able to screen 80% of their adult population for HCV infection and link most of the diagnosed cases to care and treatment, thus paving the way for the country to achieve hepatitis C elimination targets by 2030. The authors highlight the need to use all available viral detection diagnostic tools, including HCV cAg and POC HCV RNA tests, for the success of these global programs.

The article by Reipold and colleagues [15] describes the current state of POC nucleic acid detection methods, the advantages and limitations of currently available assays and the potential of nucleic acid detection–based assays in transforming diagnostics, including the challenges that would need to be addressed for wider adoption of these technologies in clinical practice. The authors examine various types of POC assays for detection of viral nucleic acids and delineate the differences between “near POC” and “true POC” assays. Several state-of-the art technologies that have a promising future in HCV diagnostics are also discussed.

In their article on opportunities for enhanced prevention and control of hepatitis C through improved screening and testing, Cartwright and Patel [16] discuss the importance of complete, accurate, and efficient hepatitis C testing approaches for achieving hepatitis C elimination goals. They describe the current state of hepatitis C epidemiology, screening, and diagnosis in the United States and further examine various strategies to improve HCV testing. The need to move beyond the current 2-step HCV testing sequence and the inclusion of viral detection assays, the HCV cAg test, and POC HCV RNA assays in hepatitis C diagnostic algorithms is also discussed in the article.

The article by Johnson and colleagues [17] provides a perspective from public health laboratories in the United States in terms of maximizing reflex HCV RNA testing for antibody-positive samples. The authors describe results from a survey conducted by APHL in 2021 of its 99 member laboratories and the outcome of a virtual HCV diagnostic meeting hosted by APHL and CDC in 2021. The need for completion of the current 2-step testing algorithm (testing all anti-HCV reactive samples for HCV RNA) and development of a single-step algorithm for HCV diagnostics in future are further discussed.

The final article by Fricker et al [18], while examining the tools needed to support same-day diagnosis and treatment of current HCV infection, highlights the challenges with the current multiday diagnosis and treatment, which result in the loss of a number of patients who fail to receive treatment. Current practices, diagnostic tools, and gaps in care in the field of hepatitis C in the United States are also discussed, in addition to the steps that need to be taken for same-day testing and care are discussed. The authors emphasize the need for new tools, such as FDA-approved, CLIA-waived POC antigen tests or NATs for HCV and hepatitis B virus and NATs for human immunodeficiency virus that do not require venous blood; the availability of such tools will bring a paradigm shift in our approach to achieving the goal of same-day testing and treatment.

Eliminating hepatitis C as a public health threat in the United States is achievable but requires unfettered access to early diagnosis and timely treatment [19]. A transformative national hepatitis C elimination program has been proposed by the highest levels of US government [20]. One of the three priorities identified in the proposed national elimination initiative is to accelerate the availability of innovative hepatitis C diagnostic tests, including POC testing supporting same-day testing and treatment. This 8-part supplement characterizes advancements in hepatitis C diagnostics and recent changes in the US regulatory landscape that, together, provide opportunities to expand patient-centered hepatitis C testing and treatment services across the variety of settings where people with hepatitis C receive care. Identifying and seizing these opportunities will be essential to eliminating hepatitis C as a public health threat in the United States.

## Notes

***Supplement sponsorship.*** This article appears as part of the supplement “Towards a Single-Step Diagnosis of Hepatitis C Virus Infection,” sponsored by the Centers for Disease Control and Prevention.
